# Lectures on Ectopic Pregnancy and Pelvic Hæmatocele

**Published:** 1889-03

**Authors:** 


*Lectures on Ectopic Pregnancy and Pelvic Hæmatocele.* By LAWSON TAIT, F.R.C.S. Pp. 107. Birmingham: THE "JOURNAL" PRINTING WORKS. 1888.

It is characteristic of all Lawson Tait's work that it bears the visible impress of a strong and independent personality, and that it goes straight towards some definite practical end, in complete disregard of preconceived theories or notions, and often through the ruins of them.

We regard Mr. Tait's practical work in the treatment of one phase of ectopic pregnancy as his best and greatest; and we have little hesitation in affirming that the future surgical historian will place it so. It is probable, perhaps certain, that more elaborate, learned, and even scientific treatises on the subject have been, and will be, written; but we doubt if any single man will ever again be able, in this walk of surgery, to speak of so many lives saved by operations which were as original as they were daring.

If we smile at, we can sympathise with the impatient contempt with which the practical man, dealing with the great issues of life and death, sweeps away the theories of the dissector and the student dealing with pickled specimens and inherited lore. No one has a better right to do this than Mr. Tait, and we must do him the justice to say that he makes full use of it. From the primal selection of its ectopic spot to the final death of the fully developed fœtus, there is not a question as to anatomy, diagnosis, or treatment in which Mr. Tait does not upset an opponent, or maintain an independent plan of treatment. The work is controversial throughout: it was bound to be so, for the proud position of Mr. Tait's thesis is established on the confutation of accepted doctrines. The two real strongholds of his position—the locality of the ectopic nidus of the ovum, and the necessity of operating at once on primary rupture of the sac—Mr. Tait has already fully established. Not content with establishing these positions, he attacks the enemy at all points, and if they refuse always to evacuate their strongholds, they must be conscious of feelings of considerable discomfort from the battering they have received.

An adequate idea of the contents of the book could scarcely be given even in a long summary; and such summaries, at their best, are nearly always unjust to the author. To learn its lessons our readers must read the work. It will repay the trouble.

